# Complete and draft genome sequences of *Salmonella* Wandsworth and *Salmonella* Stanley isolated from insect-based food products

**DOI:** 10.1128/mra.01019-24

**Published:** 2025-03-25

**Authors:** Jennie Fischer, Maria Borowiak, Hendrik Frentzel, Beatrice Baumann, Angelina Groger, Carlus Deneke, Istvan Szabo, Marina C. Lamparter

**Affiliations:** 1 German Federal Institute for Risk Assessment (BfR), Department of Biological Safety, Food Microbiology, Host-Pathogen-Interactions, Berlin, Germany; 2 German Federal Institute for Risk Assessment (BfR), Department of Biological Safety, National Study Centre for Sequencing in Risk Assessment, Berlin, Germany; 3 German Federal Institute for Risk Assessment (BfR), Department of Biological Safety, Bacterial Toxins, Food Service, Berlin, Germany; University of Maryland School of Medicine, Baltimore, Maryland, USA

**Keywords:** *Salmonella *Wandsworth, *Salmonella *Stanley, insects, food

## Abstract

Detection of *Salmonella* in insects is rarely described, especially in insect-based food stuff. Here, we report the genome sequences of two *Salmonella* isolates belonging to *Salmonella enterica* subsp. *enterica* serovar (*S*.) Stanley and *S*. Wandsworth isolated from a ready-to-eat insect-based food sample (whole dried salted crickets), imported to Germany.

## ANNOUNCEMENT

Insects are accepted as a novel food in Europe in terms of Regulation (EU) 2015/2283 and are gaining increasing importance as an alternative food source. Studies aiming to assess food safety of novel foods are increasingly required and carried out. A study from 2022 on the microbiological contamination of 73 food products with insects or other arthropod ingredients revealed the presence of two *Salmonella* serovars in a food sample composed of whole dried salted crickets. The *Salmonella* isolation procedure followed the ISO 6579-1:2017 as previously described (1).

Detected *Salmonella* serovars Wandsworth (isolate 21-SA00706-0) and Stanley (isolate 21-SA00706-1) are seldomly isolated in Germany and the EU (1–4) but are frequently described in Southeast Asian countries and China (4–9). The significance of both serovars as foodborne pathogens is underlined by their involvement in multi-country/multi-state outbreaks (10–13).

For genomic characterization, we applied both short-read Illumina and long-read Oxford Nanopore Technologies sequencing. One single colony per isolate was enriched in lysogeny broth for 18 h at 37°C ± 1°C. The PureLink Genomic DNA mini kit was used for gDNA isolation (Invitrogen, Carlsbad, CA, USA) as a starting point for both sequencing experiments. DNA isolation and sequencing kits were used according to the instructions of the manufacturers. Default parameters were used for all software tools unless otherwise specified.

Library preparation for short-read sequencing was carried out using the Illumina DNA Prep (M) Tagmentation kit (Illumina, San Diego, CA, USA), and the libraries were sequenced on the Illumina NextSeq benchtop sequencer using the NextSeq 500/550 midoutput kit v2.5 (300 cycles, Illumina) in 2 × 149 bp cycles. Short-read sequence data were processed using the AQUAMIS v1.2.0-18-g1317d15 pipeline implementing fastp v0.19.5 for trimming (14, 15).

Library preparation for the Oxford Nanopore MinION technology (Oxford, UK) was carried out using the Rapid Barcoding Kit 96, SQK-RBK110.96 (Oxford Nanopore Technologies, Oxford, UK).

The MinION libraries were sequenced for 48 h on an ONT MinION Mk1C device using a MinION R9.4.1 (FLO-MIN106) flowcell. Basecalling was performed on a graphics processing unit (GPU) server using a guppy version 6.4.8 in SUP mode (https://community.nanoporetech.com/downloads). The reads obtained were trimmed using Porechop v0.2.4 (https://github.com/rrwick/Porechop), filtered using NanoFilt v2.8.0, and quality checked using NanoStat v1.5.0 (16). For assembly of the genome sequences, long-read data were subjected to flye v2.9 (17). Flye-generated (closed) genome assemblies were polished in five iterations with Illumina short-read data using pilon v1.24 (18). Genome sequences were analyzed in Geneious Prime v2023.2.1. All details about read quality control and assembly are summarized in Table 1. Annotation was performed by NCBI PGAP v6.7 (19). The BakCharak pipeline v3.1.2-2-g8ac6c3c was further used for in-depth characterization of isolates regarding antimicrobial resistance, plasmid, and virulence markers (https://gitlab.com/bfr_bioinformatics/bakcharak) (Table 1). Remarkably, PHASTER analysis (20) revealed that a 92,749 bp circular contig shared by both genomes exhibits characteristics consistent with phage genomes (contigs confirmed in PFGE Analysis, data not shown). Genome characterization and data accessibility for these two *Salmonella* strains are crucial to better understand potential contamination routes in insect-based food production lines, facilitating risk assessment for this emerging food source.

**TABLE 1:** Genetic features of *Salmonella* Wandsworth and *Salmonella* Stanley isolated from insect-based food products

	*S*. Wandsworth 21-SA00706-0	*S*. Stanely 21-SA00706-1	Tool, database, and settings
Short-read quality control	1,960,830 reads; 86.7% ≥ Q30	2,086,436 reads; (87.0% ≥ Q30)	AQUAMIS v1.2.0-18-g1317d15 (14) utilizing fastp v0.19.5 (15)
Long-read quality control	44,361 reads; N50: 9,630 bp	38,444 reads; N50: 8,718 bp	Porechop v0.2.4 (https://github.com/rrwick/Porechop) NanoFilt v2.8.0 & NanoStat v1.5.0 (16)
Assembly	GCF_037099745.1 (complete, two contigs) NZ_CP146847.1: chromosome; 4,793,104 bp; circular; %GC: 52.2 NZ_CP146848.1: p21-SA00706-0; 92,749 bp; circular; %GC: 49.2	GCF_037083705.1 (draft, three contigs) NZ_JBAPCC010000001.1: chromosome; 4,927,615 bp; linear; %GC: 52.2 NZ_JBAPCC010000002.1: unknown; 37,811 bp; linear; %GC: 48.5 NZ_JBAPCC010000003.1: p21-SA00706-1; 92,749 bp; circular; %GC: 49.2	Flye v2.9 (17) pilon v1.24 (18) Geneious Prime v2023.2.1
MLST	1498	29	BakCharak pipeline v3.1.2-2-g8ac6c3c (https://gitlab.com/bfr_bioinformatics/bakcharak) utilizing MLST v2.23.0 (https://github.com/tseemann/mlst) & PubMLST Database: 2023-01-15
Plasmid marker	None	None	BakCharak pipeline v3.1.2-2-g8ac6c3c utilizing abricate v1.0.1 (https://github.com/tseemann/abricate) & PlasmidFinder Database: 2021-03-27 (21)
Antimicrobial resistance genes (identity)	mdsA (98.3%); mdsB (99.6%)	mdsA (98.3%); mdsB (99.6%)	BakCharak pipeline v3.1.2-2-g8ac6c3c utilizing NCBI AMRFinderPlus v3.12.8 & AMRFinder Database: 2024-01-31.1 (22, 23)
Virulence gene count by class	Adherence (25); antimicrobial activity/competitive advantage (2); effector delivery system (92); immune modulation (1); invasion (2); motility (1); nutritional/metabolic factor (7); regulation (5)	Adherence (25); antimicrobial activity/competitive advantage (2); effector delivery_system (87); immune modulation (2); invasion (2); motility (1); nutritional/metabolic factor (12); regulation (5)	BakCharak pipeline v3.1.2-2-g8ac6c3c utilizing abricate v1.0.1 & db VFDB_all_setA Database: 2022-08-26 (24, 25)
Closest NCBI reference	None detected	None detected	NCBI Pathogen Detection Isolate Browser, 24-05-31 (https://www.ncbi.nlm.nih.gov/pathogens/isolates//#)

## Data Availability

All data are encompassed under BioProject number PRJNA937468. Sequencing raw reads of *S*. Wandsworth 21-SA00706-0 (BioSample: SAMN40213193) were deposited in the NCBI Sequence Read Archive (SRA) (accession numbers SRR28174165 (ONT data) and SRR28174166 (Illumina data). The complete genome sequence is available at NCBI RefSeq assembly: GCF_037099745.1. Sequencing raw reads of *S*. Stanley 21-SA00706-1 (BioSample: SAMN40213194) were deposited in the NCBI SRA (accession numbers SRR28174163) (ONT data) and SRR28174164 (Illumina data). The draft genome is available at NCBI RefSeq assembly: GCA_037083705.1.
